# On the electrical conductivity of alginate hydrogels

**DOI:** 10.1093/rb/rby019

**Published:** 2018-08-13

**Authors:** Georgia Kaklamani, Diana Kazaryan, James Bowen, Fabrice Iacovella, Spiros H Anastasiadis, George Deligeorgis

**Affiliations:** 1 Institute of Electronic Structure & Laser, Foundation for Research & Technology Hellas, P.O. Box 1385, Heraklion, Crete, Greece; 2 School of Engineering and Innovation, The Open University, Milton Keynes, UK and; 3 Department of Chemistry, University of Crete, P.O. Box 2208, Heraklion, Crete, Greece

**Keywords:** alginate, bioelectronics, conductivity, hydrogel, polysaccharide, resistance, fibroblasts

## Abstract

Hydrogels have been extensively used in the field of biomedical applications, offering customizable natural, synthetic or hybrid materials, particularly relevant in the field of tissue engineering. In the bioelectronics discipline, hydrogels are promising mainly as sensing platforms with or without encapsulated cells, showing great potential in healthcare and medicine. However, to date there is little data in the literature which characterizes the electrical properties of tissue engineering materials which are relevant to bioelectronics. In this work, we present electrical characterization of alginate hydrogels, a natural polysaccharide, using a four-probe method similar to electrical impedance spectroscopy. The acquired conductance data show distinct frequency-dependent features that change as a function of alginate and crosslinker concentration reflecting ion kinetics inside the measured sample. Furthermore, the presence of NIH 3T3 fibroblasts encapsulated in the hydrogels matrix was found to alter the artificial tissue’s electrical properties. The method used provides valuable insight to the frequency-dependent electrical response of the resulting systems. It is hoped that the outcome of this research will be of use in the development of cell/electronic interfaces, possibly toward diagnostic biosensors and therapeutic bioelectronics.

## Introduction

Hydrogels are three-dimensional polymeric networks crosslinked via chemical bonds, ionic interactions, hydrogen bonds, hydrophobic interactions or physical bonds [1]. They are highly hydrophilic, able to absorb large amount of water [2]. Due to their hydrophilic nature and their similarity to the extracellular matrix (ECM), hydrogels are also predominantly biocompatible providing a low immunogenic reaction risk [3]. Furthermore, cells can be easily encapsulated, and the resulting gel can be highly functional *in vivo* and *in vitro* [4, 5].

Hydrogels characteristics such as physical, chemical and mechanical properties are strongly dependent on the type of the polymer used for their production that can be natural, synthetic or semi-synthetic material [6]. The material selection stems from the fact that the resulting hydrogels possess similar water content and have similar elastic moduli very close to the body tissues making them biocompatible *in vivo* and *in vitro*. Hydrogels have many applications in the biomedical field, specifically in regenerative medicine. They can be used as scaffolds due to the structural stability they offer, as drug delivery systems, as cell or peptides delivery agents, as biosensors or just as barriers in order to provide stabilization and prevent infection [7].

Biosensor platforms are increasingly being developed for use in point-of-care diagnostics [8], drug delivery and environmental monitoring [9]. Specific applications include pathogen detection [10], monitoring glucose concentration in tears [11] and rapid detection of human papilloma virus [12].

Specifically, the biosensing device should have minimal impact to the body, should be biocompatible and non-toxic [13, 14]. A biosensing receptor should be able to receive a chemical or physical stimulus and transform it into a form of energy that the transducer is able to process into a comprehensible analytic signal that can be received, processed and studied. Such signals are typically electrical. Hydrogels are used in biosensing as stabilizing agents since they offer a stable environment to the immobilized cells, or the hydrogel itself may act as the biosensor [15]. The hydrophilicity of the hydrogels plays a crucial role in their capacity as biosensors since it is a key characteristic toward their biocompatibility and provides facile conductivity through dissolved ions [16].

Electroconductive hydrogels belong to the general class of multifunctional smart materials, as they can react to specific environmental changes [17]. An electroconductive hydrogel should have groups that create charge mobility throughout their body. Some conductive hydrogels contain conjugated systems where the chemical bonding leads to an unpaired electron (π electron) per carbon atom. This π electron then moves along the backbone of the ring due to the electron delocalization that is created because of the overlap of the orbitals that π bonding occurs. The electron delocalization creates the charge mobility along the polymer chain [18]. Other groups are ionic groups, i.e. -COO-, that help in the movement of the electrons throughout the chains. Furthermore, hydrogels present a class of high water content polymers with physical or chemical crosslinking. As a result of the water content most hydrogels also present conductivity due to the water self-ionization process that creates constant ionic flow in the hydrogels. Finally, in case the nature of the hydrogel allows ionic crosslinking to occur, then an extra set of ions is induced. Due to the presence of those ions inside the gel, the gel’s conductivity is expected to increase. This effect strongly depends upon the degree of crosslinking [19].

Alginate is a naturally occurring polysaccharide extracted from brown algae such as Laminaria hyperborea and lessonia that are found in coastal waters around the globe. Alginate is a linear unbranched copolymer that contains homopolymeric blocks of (1,4)-b-d-mannuronic acid (M) and a-l-guluronic acid (G), which are covalently linked together in different sequences or blocks. The amount of each depends on the source of the alginate [17]. The block sequence can either be the same or alternating. Each type of monomer offers different properties to the hydrogel. The formation of the gel is a result of ionic crosslinking agents, like multivalent cations (Ca^2+^, Sr^2+^, Ba^2+^) that form ionic inter-chain bridges between G-blocks of alginate chains. Due to the linkage of the G-blocks, a hydrogel that has a higher ratio G/M is stiffer than a hydrogel with a low G/M ratio [20, 21]. If the G/M ratio is small, then the gel exhibits higher porosity. Those characteristics play an important role in the structure and the final use of the gel. Furthermore, the size of the pores affects the flow of the nutrients throughout the gel, as well as the vascularization process, that is crucial to tissue regeneration in tissue engineering applications.

Alginate is biocompatible with the mammalian skin, non-toxic and non-immunogenic [22]. Also, alginate-based hydrogels gel under mild conditions, therefore they are used for the encapsulation of various substances with little trauma. However, due to their hydrophilic nature, alginate-based hydrogels show poor adhesion to mammalian cells [23], thus they do not interact directly with the cells. Cell anchorage to the ECM plays a crucial role, thus alginate hydrogels are often modified with simple chemistries, such as RGD-peptides [24]. The existing carboxyl groups of the polymers give the ability to covalently modify the gel. This property makes them eligible for tissue engineering applications. Finally, alginate gels do not degrade naturally by mammalian enzymes [25]. Their degradation occurs because the Ca^2+^ ions slowly diffuse out of the gel. If the hydrogel is used as a biosensing agent this is an advantage, offering a long degradation time. The hydrogel acts as a stable support for the encapsulated cells that work as sensors for various macromolecules.

Electrical impedance as a function of frequency—called electrical impedance spectroscopy (EIS)—can reveal details regarding the conduction mechanism in a complex system such as the hydrogel. Electrical resistivity or ionic conduction in ionic conducting polymers, infused salts and liquid or solid electrolytes is frequently studied using impedance spectroscopy [26]. Using EIS, the cellular responses in bulk artificial and biological tissues have been studied [27]. The development of cell layers [28], the electrical properties of porcine tissues [29], the impedance of tofu [30], the impedance of viable zebra fish embryos [31] have been studied and monitored with this technique.

In EIS, charge transfer between two electrodes and an electrolyte is studied by applying a frequency varying electric stimuli [32]. Typically, a potential difference is applied between two electrodes and the electrical current is measured. In EIS the response of the sample under test can be described by its impedance *Z* = *Z*′ + *iZ*″’, where *Z*′ is the resistance and *Z*″ is a reactive term describing the delay in ion movement in the system. Measuring the amplitude and phase difference provides information pertinent to the speed at which ions move in the conducting sample. Unfortunately, this relationship is affected by many parameters that determine the measured resistance. Parameters that can influence the measurements are not only bulk resistive-capacitive effects but also electrode reactions such as adsorption or chemical modification due to reactive ion species and other. To partially mitigate reactive effects, inert Pt electrodes are frequently used to minimize the chemical interaction between sample and electrodes. However, the resistance caused by the probes is still an important issue that masks the studied sample behaviour.

The aim of this work was to investigate the conducting properties of alginate-based hydrogels as a function of crosslinker, alginate concentration and the presence of cells. Furthermore, different crosslinker ions were also studied. The external gelation method was employed to produce hydrogel samples. This work aims at providing a better understanding of the electrical properties of hydrogels, which can be used in the design and application of such materials for bioelectronics. It is also believed that the electrical properties of such artificial tissues could provide a ground base in biosensing applications in terms of understanding the effect of electrical stimuli on the behaviour of the encapsulated cells.

## Materials and methods

### Materials

All chemicals were sourced from Sigma Aldrich (UK) unless otherwise stated. Purities were >99% in all cases. HPLC-grade H_2_O was employed throughout all experiments.

### Overview

To study the electrical properties of the alginate hydrogels, a series of hydrogels were fabricated with varying crosslinker ions (Ca^2+^ and Sr^2+^), crosslinker concentration (1, 2 and 5 M) and sodium alginate (NaAlg, product code 180947) concentration (1, 2 and 5%). Finally, the hydrogel properties with cell culture medium and hydrogels encapsulated with 3T3 fibroblasts were also measured. All hydrogels were electrically characterised using the setup described in ‘Electrical characterisation’ section to provide an insight of electrical performance as a function of fabrication parameters.

### Hydrogel preparation

Hydrogels (HGs) were prepared using a previously reported external gelation method [17]. For the sake of completeness, the process is briefly described here: NaAlg powder was dissolved in H_2_O under agitated conditions at a temperature of 70°C and stirred for a minimum of 2 h using a hotplate stirrer. The concentrations of NaAlg solutions were 1.0% (w/v), 2.0% (w/v) and 5.0% (w/v). Aqueous solutions of divalent cations (DCs) included calcium chloride (CaCl_2_) of molecular weight 110.98 g/mol at concentrations 1, 2, and 5 M and strontium chloride hexahydrate (SrCl_2_) of molecular weight 266.62 g/mol at concentrations 1 and 2 M. It was not possible to prepare a 5 M SrCl_2_ solution due to the limited solubility of the salt in water.

**Figure 1.**
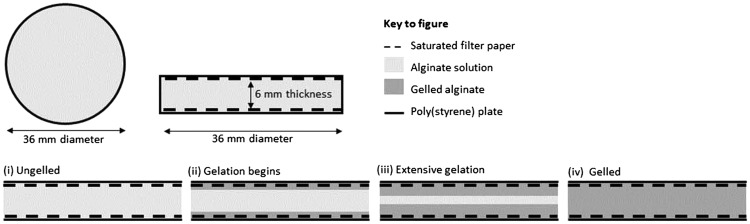
Gel geometry (top row) and gelation schematic (bottom row) showing (i) ungelled alginate solution, (ii)–(iii) progression of the gelation through the alginate solution as the cations diffuse into the alginate solution, generating crosslinks and (iv) the final, gelled alginate.

The NaAlg solution was poured into a poly(styrene) mould (90.6 mm inside diameter, 9.0 mm inside height, Sterilin, UK) to a liquid height of 6 mm and allowed to gel in the presence of an aqueous DC solution, held at the upper and lower boundaries by porous microcellulose sheets (QL100, Fisherbrand, UK) that were trimmed to match the shape of the poly(styrene) mould, and were immersed in the aqueous DC solution for 5 min immediately prior to use. The upper sheet was held in place from above using a poly(styrene) support, under a compressive load in order to maintain close contact since some shrinkage was observed at the sample edges. Samples were allowed to gel at 18°C for 60 min. The gelation geometry and schematic are shown in Fig. 1.

### Hydrogel preparation using cell growth media

A 2% (w/v) solution of NaAlg in the presence of supplemented Dulbecco’s modified Eagle’s medium (S-DMEM) was prepared. S-DMEM consists of Dulbecco’s modified Eagle’s medium (DMEM) supplemented with 2.4% (w/v) l-glutamine, 2.4% (w/v) 4-(2-hydroxyethyl)-1-piperazine ethanesulfonic acid (HEPES) buffer, and 1% (w/v) penicillin/streptomycin. Equal volumes of a 4% (w/v) NaAlg solution and S-DMEM were mixed together to create the 2% (w/v) NaAlg S-DMEM solution. The 4% (w/v) NaAlg solution was manufactured according to the procedure described above.

### Hydrogel preparation using cellular media

3T3 fibroblasts were maintained in an incubator at 37°C under an atmosphere of 5% (v/v) CO_2_ and 100% relative humidity; media were exchanged every 72 h. Cells were passaged using 0.25% (w/v) trypsin-EDTA solution on the seventh day after culture in S-DMEM, upon reaching approximately 70% confluency. A 4% (w/v) NaAlg solution and DCs solutions were each autoclaved at 121°C for 15 min prior to encapsulation of the fibroblasts. Solutions were allowed to cool to the room temperature (21°C) prior to use. It should be noted that autoclaving has been reported to reduce the viscosity of NaAlg solutions [33] although is phenomenon is outside the scope of this study. Further, sterilization of NaAlg hydrogels can be achieved using EtOH or scCO_2_ [34, 35]. The concentration of 3T3 fibroblasts used for encapsulation was 5 × 10^5^ cells/ml. All cell encapsulated samples were left to gel for 60 min prior to any characterisation.

To examine the presence of the cells inside the hydrogel the samples were tested under an optical microscope. Moreover, to determine cellular viability post encapsulation, 1 mm thickness sections were taken from the centre of the HG using a sterile blade. The sections were immersed in 0.2 μl calceinacetoxymethylester (calcein AM) for 15 min and 2.5 ml propidium iodide (PI) for 5 min, in S-DMEM at 37°C. All samples were visualized using fluorescence microscopy, employing 490 nm wavelength light for excitation.

A metabolic activity assay (MTT) was conducted to quantify the number of viable cells inside the hydrogel matrix. A tetrazolium assay was performed straight after the gelation process. The colorimetric assay employs the metabolic activity for viable cells to convert the tetrazolium dye to formazan. Briefly, the cell encapsulated hydrogels were immersed in an MTT (3-(4, 5-dimethylthiazol-2-yl)-2, 5-diphenyltetrazolium bromide) solution of concentration 1 mg/ml. The gels were incubated at 37°C under conditions of 5% (v/v) CO_2_ and 100% relative humidity overnight. The incubation allows the tetrazolium ring in the salt to cleave through the action of mitochondrial dehydrogenases, forming purple formazan crystals. After incubation, these crystals were dissolved in HCl/isopropanol solution of concentration 100 μl/ml and subjected to shaking for 30 min at RT. The absorbance of the solution was measured using a spectrophotometer (ASYS, Hitech GmbH) at 595 nm and compared with a standard absorbance curve. For this study three cell-encapsulated hydrogels were prepared and tested. The results provided an estimation of the cell density inside the hydrogels.

### Electrical characterisation

**Figure 2.**
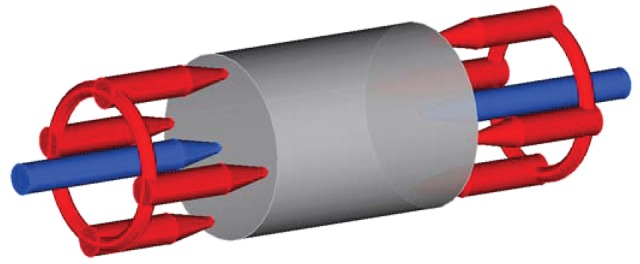
The four-probe setup realised in this work. The central electrodes (marked with blue) are the sense electrodes to measure sample voltage. The red electrodes are the force electrodes (multiple on each side) to apply the stimuli in a uniform manner. The grey cylindrical shape corresponds to the actual sample being measured.

In this work, we have realised a four-probe measuring setup to further reduce the effect of electrode resistance to the studied hydrogels. In such a system the force electrodes are spatially separated from the sense electrodes as shown in the schematic of Fig. 2. This configuration provides more accurate voltage sensing on the sample thus bypassing the contact resistance of the electrodes through which the current is actually channelled. It should be noted that in this work the amplitude of the impedance is measured.

**Figure 3.**
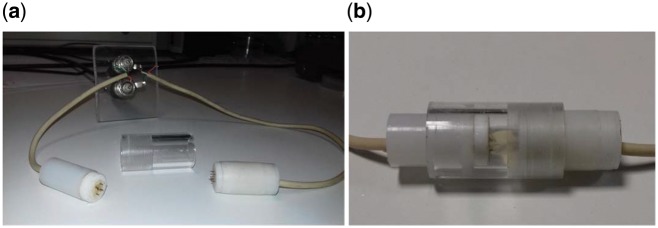
Detailed view of the four-point-probe and cylindrical sheath: (**a**) 10 stainless steel electrodes, the external electrodes provide current, whereas the central electrodes measure the voltage; the sheath minimises dehydration losses; (**b**) alginate hydrogel (with lateral dimensions 10 mm × 15 mm) placed in the R-cell prior to measurement.

The electrical properties of NaAlg HGs were measured immediately after manufacture. The experimental setup used is shown in Fig. 3a. Each sample was cut to lateral dimensions 10 mm × 15 mm and placed with the smaller face against the one side of the four-point-probe resistance cell, hereafter referred to as the R-cell, is shown in Fig. 3. The R-cell consists of two lids, each of which presents five needle-type electrodes; four electrodes conduct the current through the HG, and one electrode measures the system voltage. All electrodes penetrated approximately 1 mm into the gel to ensure electrical contact. Finally, the hydrogel was covered by a cylindrical acrylic sheath—shown in Fig. 3b—to prevent gel dehydration. Voltage and current were controlled by a double SMU Keithley 2604B. Initially, measurements were performed at a fixed frequency of 10 Hz over the current range 10^−6^–10^−2^a minimum of four times for each current setting. Subsequently, the minimum current at which good measurement repeatability was achieved was selected and the frequency was swept between 0.01 and 20 Hz. For each measurement, multiple cycles (50–100 cycles) were used to measure the voltage on the sense electrodes and calculate the hydrogel resistance. The process was automated using a custom-built computer control software.

## Results and discussion

The results present the measured conductance of the HG samples. The variables explored include NaAlg concentration, DCs concentration, current intensity, AC frequency, the presence of S-DMEM and the encapsulation of cells. For the cell-encapsulated hydrogels, optical microscopy, fluorescence microscopy and MTT assay were also conducted to examine the presence and the viability of the cells as well as the approximate cell number inside the alginate hydrogels.

### Hydrogel current stability

**Figure 4.**
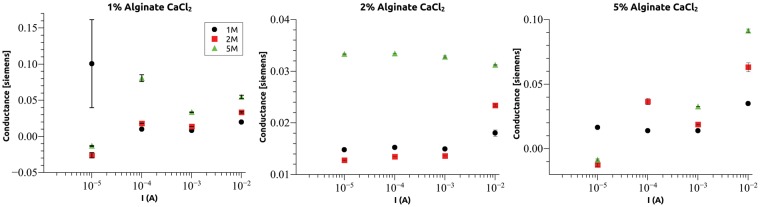
Conductance of NaAlg hydrogel crosslinked using Ca^2+^ ions as a function of current. For each data point, four measurements were performed. Error bars are smaller than the size of the datapoint for all currents larger than 10^*−*^^4 ^A. Lower current limit of 10^*−*^^5 ^A is set by the measurement setup noise floor and upper limit was attributed to gel stability. The three plots correspond to varying alginate concentration of 1% (left), 2% (centre) and 5% (right). In each plot, the three datasets (black, red and green) correspond to varying CaCl_2_ concentration. All measurements were performed using a 10 Hz stimulation frequency.

Initially each hydrogel was measured as a function of current density using AC (10 Hz) stimuli. For each current level, a single hydrogel was used, and the measurement was repeated four times. This method was chosen to remove variability due to hydrogel size. As shown in Fig. 4, at a current level of 10^−5 ^A that corresponds to a current density of approximately 1.6 × 10^−5^A/cm^2^ the measurement repeatability is affected as shown by the increased error bars in the conductivity measurements. That response was attributed to the detection noise of the setup and not to the hydrogel behaviour and is a subject of further improvement. Above 10^−4 ^A the measurements were grouped to a tight margin. The hydrogel conductivity shows a large range of constant values ranging from the lower limit up to 10^−2 ^A (1.6 × 10^−2^A/cm^2^). Above those current density values, the conductivity of the hydrogel exhibited gradual degradation over multiple measurements signalling permanent damage due to the increased current flowing through the system. This is evident in Fig. 4 from the increase of the conductance compared with lower current values in most cases.

**Figure 5.**
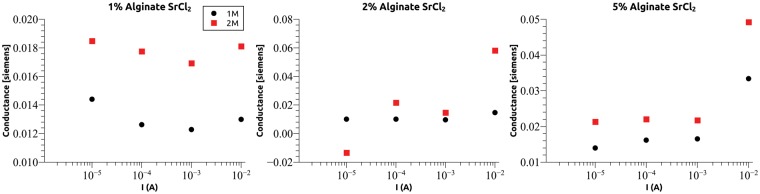
Conductance of NaAlg hydrogel crosslinked using Sr^2+^ ions as a function of current. Again the depicted datapoint is an average of four measurements and error bars are too small to show on the scale. Lower limit is set by the measurement setup noise floor and upper limit was attributed to gel stability. The three plots correspond to varying alginate concentration of 1% (left), 2% (centre) and 5% (right). In each plot the two datasets (black and red) correspond to two different SrCl_2_ concentration. All measurements were performed using a 10 Hz stimulation frequency.

Similar results were obtained for the case were SrCl_2_ was used as a crosslinker shown in Fig. 5. Hydrogel conductance exhibited the tendency to increase slightly with increasing cation concentration. This is to be expected as higher ion concentration provides larger charge density in the gel. There does not appear to be any systematic dependence on NaAlg concentration. The conductance of hydrogels crosslinked with Ca^2+^ was larger than those crosslinked using Sr^2+^. Assuming there is no significant difference in charge concentration between the two cases, the cation size (Sr radius is 255 pm while Ca radius is 231 pm) is expected to affect cation mobility in the polymeric matrix that is formed by the alginate. This could explain the observed dependence. The current used in subsequent measurements was fixed at 10^−4 ^A as this was the smallest value that produced repeatable conductance measurements.

### Hydrogel conductance as a function of frequency

**Figure 6.**
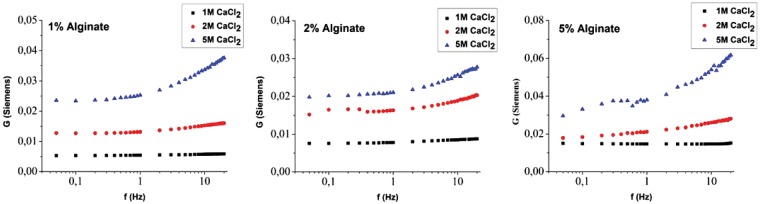
Hydrogel conductance as a function of AC frequency, at a current intensity of 10^*−*^^4 ^A for 1% (w/v) NaAlg (left) 2% (w/v) NaAlg (centre) and 5% (w/v) NaAlg (right). All cases were crosslinked with Ca^2+^ at concentration from 1 to 5 M as shown in the legend.

Conductance for a fixed current (10^−4^A) as a function of frequency was measured for various CaCl_2_ crosslinker and NaAlg concentrations. Figure 6 shows the resulting conductance grouped by NaAlg concentration. In all cases, higher stimulation frequency corresponds to higher conductance.

Furthermore, in all cases the low crosslinker concentration exhibits minimal frequency dependence whereas the high crosslinker concentration exhibits large conduction change between the low (10 mHz) and high (20 Hz) values. Inside the hydrogel there are different types of free ions such as the ions produced by the dissociation of water, OH^−^ and H_3_O^+^, as well as Cl^−^ and the DCs Ca^2+^ or Sr^2+^. Na^+^ ions are also present from the dissociation of NaAlg upon dissolution. The cations are incorporated in the intermolecular bridges between carboxylic acid moieties, which are formed during the gelation process. All these ions may contribute to the ionic flow within the HG, which contributes to the conductance. The hydrogel structure is also known to strongly depend on crosslinker concentration with higher concentrations resulting in shorter chains between crosslinked sites. This translates to a more compact structure that results in increased stiffness [17]. Ion movement is directly affected by the stimulation frequency with higher frequencies corresponding to shorter and faster oscillatory movement of ions inside the hydrogel matrix. It is thus expected that a large movement will eventually be hindered by the polymeric chains and thus translate to lower conductance. The effect is as expected more pronounced by the dense polymeric matrix in the high crosslinker content hydrogels.

At high NaAlg and high crosslinker concentration, a secondary plateau is observed around 0.5 Hz. This effect needs further study and could potentially correlate to structural changes in the hydrogel.

**Figure 7.**
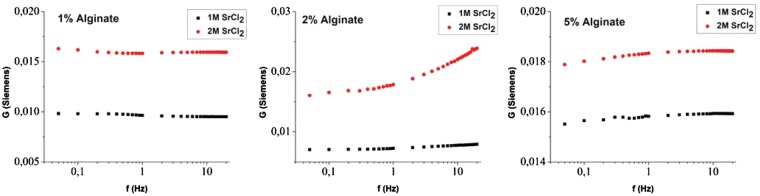
Hydrogel conductance as a function of AC frequency, at a current intensity of 10^*−*^^4 ^A for 1% (w/v) NaAlg (left), 2% (w/v) NaAlg (centre) and 5% (w/v) NaAlg (right). All cases were crosslinked with Sr^2+^ at concentration from 1 to 5 M as shown in the legend.

The frequency response for the case of SrCl_2_ used as a crosslinking agent is shown in Fig. 7. In this case, the distinct frequency dependence observed for the CaCl_2_ case is only visible for the 2% (w/v) NaAlg case. On the contrary the 1% and 5% show a rather flat response for the frequency range used in this study. Sr^2+^ has a larger ionic radius than Ca^2+^ and it creates different interactions during the gelation with the alginate blocks. Ca^2+^ creates ionic bridges with both G and M blocks while Sr^2+^ only interacts with G blocks. Thus, fewer ionic groups are present inside the hydrogel after the gelation. The larger radius of Sr^2+^ together with the limited interactions within the gel and the low concentration of carboxylic acid moieties results in decreased hydrogel conductance [36]. We argue that in the Sr case, the increasing alginate concentration results in increased interaction between the ions and the polymer. Effectively the measured frequency response is shifted as a function of alginate concentration and since only a limited range of frequencies is measured, a fraction of the entire curve is measured each time. Further study using a higher frequency range and phase measurement will provide insight to the mechanism inducing this response.

The electrical properties of conductive hydrogels have previously been investigated using the impedance spectroscopy technique. Alexe-Ionescu *et al.* [37] studied a hydrogel with composition of 1% propylene glycol, 2% hydroxyethyl cellulose and 97% deionised water, over the range of frequencies 0.01–20 Hz. The measured resistance decreased as the AC frequency increased, i.e. the conductance increased with increasing AC frequency, which is in agreement with our results.

### Response of hydrogel containing cell culture medium

A series of hydrogels containing S-DMEM medium—described in the experimental section—were also subjected to electrical characterization using the same technique to clarify the contribution of the cell culture medium to the total conductance.

**Figure 8.**
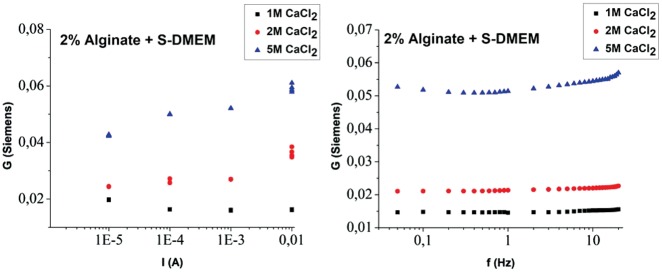
Conductance of 2% (w/v) NaAlg hydrogels containing S-DMEM, as a function of current density (left), at an AC frequency of 10 Hz and as a function of frequency (right) for 10^*−*^^4 ^A current.

In stark contrast to the NaAlg/CaCl_2_ hydrogels described in the previous section, the conductance increases significantly with increasing current as shown in Fig. 8 (left). This phenomenon could be related to the salts existing in the medium. The alginate/S-DMEM hydrogel is composed only of 2% polymer. The rest is water and salts that are very possible to be dissociated and proteins together with carbohydrates coming from the S-DMEM. Thus, the free ions that are assumed to exist in the Alginate/S-DMEM hydrogel are Na^+^, K^+^, ${\text{SO}}_{4}^{2}$^–^, Ca^2+^, Cl^–^ and Mg^2+^ [38]. The mobility of such ions is very possible to contribute to the increase of the conductance of the hydrogel. Further evidence of conductance due to the medium is provided in the frequency-dependent conductance plotted in Fig. 8 (right) were the characteristic monotonic increase of conductance with frequency is not observed.

### Hydrogels containing 3T3 fibroblasts

**Figure 9.**
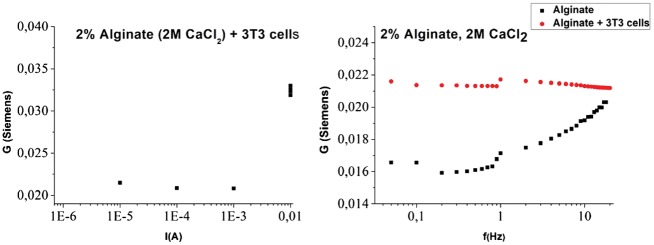
Conductance of 2% (w/v) NaAlg HGs crosslinked with Ca^2+^ at 2 M, containing S-DMEM and 3T3 fibroblasts, (left) as a function of current intensity, at an AC frequency of 10 Hz: (right) as a function of AC frequency, at a current intensity of 10^*−*^^4 ^A.

As described in the experimental section, hydrogels were also prepared incorporating 3T3 fibroblasts at a cell density of 5 × 10^5^ cells/ml. Assuming an average fibroblast volume of 2 × 10^3^ μm^3^, the fibroblasts occupy approximately 0.1% of the total hydrogel volume. Figure 9 (left) shows the conductance of the resulting hydrogel as a function of current density.

It is shown that the response is similar to the hydrogel with S-DMEM growth medium shown in Fig. 8 (left) with red datapoints. However, a distinct change is evident in the frequency dependence shown in Fig. 9 (right) comparing the two cases. The cell containing hydrogel exhibits a flat response. It is assumed that fibroblasts dominate the conductance as evident by the increase of total conductivity for all measured frequencies. Also, the conductance is attributed to much more mobile ions since the frequency dependence is flat compared with the acellular HGs.

**Figure 10.**
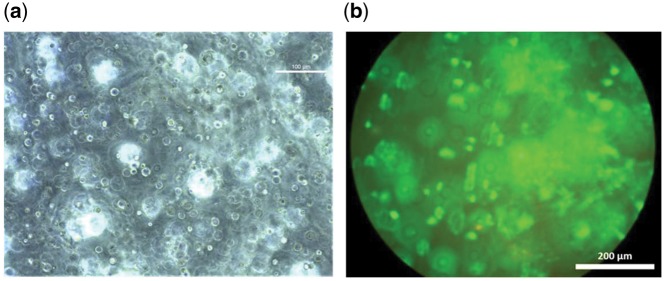
(**a**) Optical microscopy image of 2% NaAlg hydrogels crosslinked with Ca^2+^ at 2 M, containing S-DMEM and 3T3 fibroblasts immediately after gelation. (**b**) Cell viability within 2% (w/v) NaAlg hydrogels crosslinked with Ca^2+^ at 2 M, containing S-DMEM and 3T3 fibroblasts, assessed using live/dead staining immediately after gelation.

Finally, to verify the presence of the cells inside the hydrogels and the cell viability prior to electrical characterization, optical microscopy and PI/AM stain were used on the cell containing hydrogels (Fig. 10). Optical microscopy (Fig. 10a) shows the presence of cells encapsulated inside the hydrogel where the cells appear to be well dispersed. The cross-section (Fig. 10b) shows that the majority of the cells are viable, and therefore it is assumed they were alive during the electrical characterisation.

Also an MTT assay was conducted straight after the hydrogel production to quantify the cell number inside the hydrogel. Three hydrogels were tested, and the results were compared with a standard growth curve. The mean value of the approximate cell number inside the hydrogels was found at 518.000 cells/ml of sample, which is in agreement with the initial cell population (5 × 10^5^ cells/ml) that was inserted in the hydrogels.

In the literature it has been reported that cells respond to electrical stimuli [39]. This response can be translated as genes expression, cells differentiation, cells migration or guided cell growth [40–42]. Possible explanation for the cellular responses in such stimulus includes the alteration of the potential of the cell membrane, modification of ion channels and calcium channel activation [43]. The cell membrane is penetrated by ion channels which are composed of proteins. These channels act like gates allowing ions and currents to flow. The proteins existing in the cell membrane play a critical role in the fusion process [44, 45].

Several studies have shown that the adhesion, migration and proliferation of cells on 2D substrates can be strongly influenced by electrical stimuli [46], however, similar studies of cells within 3D structures are limited. Cho *et al.* have studied adhesion and orientation of mesenchymal stem cells (MSCs) and fibroblasts in 3D collagen structures while applying electrical stimuli. Their findings showed that fibroblasts responded to the stimuli by perpendicular reorientation while MSCs showed only small changes. This is attributed to the limited adhesion of fibroblasts to the collagen matrix [47]. Fibroblasts are known to interact with the ECM through focal adhesions which are integrin-based and link strongly the extracellular polymer matrix and the cells cytoskeleton [48]. Also, the adhesion of fibroblasts is facilitated through fibrillar adhesion connecting cells to the ECM using fibronectin [49]. In the case of alginate hydrogel, it has been reported that cells do not adhere, unless the hydrogel is chemically modified with peptides [24]. Also, the specific hydrogel is in general hydrophilic and inhibits the protein adsorption which favours cell adhesion [38]. In our case, the fibroblasts do not adhere inside the alginate matrix, something that is confirmed from the spherical shape of the cells shown in Fig. 10. However, it has been shown in the literature that the cation channels of the specific cell type can be activated without being connected to the ECM of other cells [24, 50–52]. Since the fibroblasts do not adhere to the polymer matrix, it is assumed that the increase of conductance is due to the activation of the cation channels, the increase of the calcium ions concentration and the mobility of other ions inside the hydrogel matrix.

The exact reasons for the cells encapsulated inside the hydrogel matrix responses are not clear enough and are currently under investigation. A variety of cell populations, together with different hydrogels composition and the encapsulation of different cells types are the focus of our future work. Finally, an optimization of our electrical setup so that it will friendly host artificial tissues under sterile conditions is under fabrication.

## Conclusions

A series of alginate-based hydrogels were fabricated using both CaCl_2_ and SrCl_2_ crosslinkers to achieve gelation for various NaAlg and crosslinker concentrations. All hydrogels electrical conductance as a function of current density and frequency were measured and were shown to exhibit characteristic differences attributed to the hydrogel structure and ion concentration. Specifically, the conductance of acellular Ca^2+^-crosslinked hydrogels was larger than acellular Sr^2+^-crosslinked hydrogels. Further, conductance increased with increasing alginate concentration and increasing concentrations of the DC. Finally, hydrogels containing cell culture medium and 3T3 fibroblasts were also characterised to explore their electrical response. It was found that distinct response attributed to the fibroblast existence even at 0.1% (v/v) cell concentration dominated the electrical behaviour of the resulting hydrogels.

Further investigations will explore the conductance of alginate solutions prior to gelation, as well as solutions of equivalent ionic strength, but free from alginate. The importance of volume fraction of inclusions will be investigated, using electrically conductive and electrically insulating inclusions, as well as cells, in order to better understand the effect of cells presence in the conductance. The technique could be potentially used to predict hydrogel electrical performance in tissue engineering and biosensing applications as well as an analytical tool to better understand ion kinetics in the presence of live tissue.

## Ethical approval

This article does not contain any studies with human participants or animals performed by any of the authors.
